# Facile control of silica nanoparticles using a novel solvent varying method for the fabrication of artificial opal photonic crystals

**DOI:** 10.1007/s11051-016-3691-8

**Published:** 2016-12-17

**Authors:** Weihong Gao, Muriel Rigout, Huw Owens

**Affiliations:** 1 School of Materials, The University of Manchester, Manchester, M13 9PL UK; 2 School of Design, University of Leeds, Leeds, LS2 9JT UK

**Keywords:** Silica nanoparticles, Solvent varying method, Artificial opals, Photonic crystals, Stöber method, Colloids

## Abstract

In this work, the Stöber process was applied to produce uniform silica nanoparticles (SNPs) in the meso-scale size range. The novel aspect of this work was to control the produced silica particle size by only varying the volume of the solvent ethanol used, whilst fixing the other reaction conditions. Using this one-step Stöber-based solvent varying (SV) method, seven batches of SNPs with target diameters ranging from 70 to 400 nm were repeatedly reproduced, and the size distribution in terms of the polydispersity index (PDI) was well maintained (within 0.1). An exponential equation was used to fit the relationship between the particle diameter and ethanol volume. This equation allows the prediction of the amount of ethanol required in order to produce particles of any target diameter within this size range. In addition, it was found that the reaction was completed in approximately 2 h for all batches regardless of the volume of ethanol. Structurally coloured artificial opal photonic crystals (PCs) were fabricated from the prepared SNPs by self-assembly under gravity sedimentation.

**Electronic supplementary material:**

The online version of this article (doi:10.1007/s11051-016-3691-8) contains supplementary material, which is available to authorized users.

**Figure:**
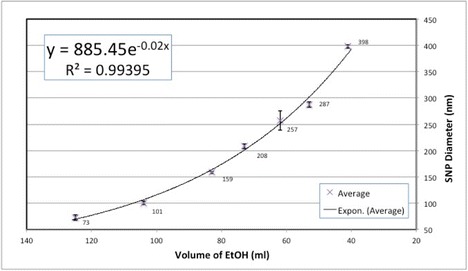
ᅟ

## Introduction

Spherical silica particles with uniform size, shape and composition have been used in various applications such as ceramics (Sacks and Tseng 1984), chromatography (Unger et al. 2000), coatings (Leder et al. 2002) and more recently in the field of photonic crystals (PCs) or colloidal crystals (Xia et al. 2000; López 2003). Natural precious opals are a typical example of PCs as they consist of highly ordered silica nanoparticles (SNPs) with diameters typically ranging between 150 and 400 nm (Jones et al. 1964; Sanders 1964; Darragh et al. 1976). Such structures exhibit a play-of-colour (POC) effect, which has inspired the fabrication of artificial opals (specifically, opal films) from the self-assembly of spherical particles (Jiang and Bertone 1999; Marlow et al. 2009; Wang et al. 2009; Galisteo-López et al. 2011). The production of high-quality opals requires a regular packing of a population of well-defined spheres with controllable size and dispersity properties.

A variety of methods of preparing uniform silica particles have been illustrated in the monograph of Iler in 1979 (Iler 1979). The hydrolysis and condensation of tetraethyl orthosilicate (TEOS) with water in the presence of ammonia as a catalyst are one commonly adopted method. Since its discovery by Kolbe (Kolbe 1956) in 1956, several studies (Stöber et al. 1968; Bogush et al. 1988; Giesche 1994; Chen et al. 1996a) have been conducted based on this reaction system. This method is known as the Stöber or SFB method, as in 1968, Stöber, Fink and Bohn (Stöber et al. 1968) developed a systematic way of controlling the silica particle diameters in the micron size range.

An important issue in preparing silica particles is the prediction of the final particle size. Using the SFB method, Bogush et al. (1988) developed an exponential equation for the prediction of particle size using the molar amounts of water, ammonia and TEOS. However, there is a 20% deviation between the predicted and experimental sizes. Santamaría Razo et al. (2008) reported a modified SFB method by mixing two prepared mother solutions: one containing ammonia-water with a fixed molar ratio and the other containing ethanol-TEOS with a fixed ethanol molar and variable TEOS molar. The final particle size is expressed using a cubic-root equation, which consists of two parameters: a constant ks which depends on the fixed molar amounts of NH_3_/H_2_O/EtOH and the molar amount of TEOS (mol_TEOS_). This equation gives a perfect fit for the particle size in the range of 200–450 nm, but there exists a large deviation (approximately 100 nm) for the particles above 450 nm. Moreover, the final particle size can be also predicted using the hydrolysis and condensation rate (Chen et al. 1996b; Kim et al. 2002) or the overall reaction rate (Giesche 1994). It should be noted that the reaction rate including hydrolysis and condensation rate is also calculated from the molar amount of the reagents. It can be concluded that the current prediction methods are all based on the molar information of the reagents, and generally, the resultant equations contain more than one parameter.

Using a seeded growth (two-step) method (Bogush et al. 1988; Chen et al. 1996a; Chen 1998) where additional TEOS is added to a prepared silica suspension, the final size can be simply expressed using one parameter: the volume amount of added TEOS, which gives an easier way to predict the particle size. Moreover, a modified seeded growth method (Kim and Kim 2002; Kim and Kim 2004; Nozawa et al. 2005) designed for a continuous production process, by the addition of a feeding pump, allows for the control of the addition rate, and it has been claimed to give greater control over the final particle size and size distribution. Essentially, the seeded growth method is a two-step process. Seed preparation and the addition of reactants extend the production time. The modification process requires additional setup and thus requires more control over the experimental equipment. In addition, until now, the prediction of the final particle size from the volume information of the seed reagent has been rarely reported.

In this work, a novel but straightforward one-step solvent varying (SV) method is proposed to control the size distribution of the Stöber silica nanoparticles for the purposes of the fabrication of artificial opal photonic crystals. Firstly, a successful reaction recipe based on the work of Pieranski [26] was selected. Varying the volume of the solvent ethanol and fixing the other reaction conditions (including the volumes of the reagents and temperature) produced several recipes. A set of seven batches of uniform SNPs were successfully produced with particle diameters ranging from 70 to 400 nm and with a polydispersity index (PDI), for all but one sample, of less than 0.1. Under these fixed conditions, it was found that the final size has a negative correlation with the volume of ethanol used in the recipe. Therefore, a simple size prediction equation containing only one parameter, the volume of ethanol, is presented. It was also determined that the chemical reaction had completed within 2 h regardless of the volume of ethanol that was used. This suggested a minimum sample preparation time and would shorten the associated production time. It should be noted that this work is concerned with the final particle size and size distribution that affects the quality of artificial opals.

## Experimental

### Chemicals and materials

The sources of chemical reagents used throughout the experiments include the following: the precursor alkoxide tetraethyl orthosilicate (TEOS) (99.0%) purchased from Sigma-Aldrich Co., LLC; the catalyst ammonia (NH_3_, 25% in H_2_O) and the solvent ethanol (EtOH, 99.9%) which were obtained from Fisher Scientific Co., Ltd., UK; the hydrolyzing agent water (H_2_O, distilled by USF-ELGA water purifier) was dispensed from the laboratory. All the materials were used as received without any further purification.

### Synthesis of uniform SNPs

Silica nanoparticles (SNPs) were synthesised based on the Stöber method (Stöber et al. 1968), where hydrolysis and condensation of silicone alkoxide were catalysed by ammonia. Firstly, a starting solution containing 83 ml of ethanol, 8 ml of ammonia (25%) and 3 ml of distilled water was prepared in a 250-ml round-bottom flask under vigorous stirring. Once the mixture reached 60 °C, 6 ml of TEOS was then added into the solution. Following the addition of the TEOS, an increasing opalescence of the mixture was observed for the following 1–5 min depending on the concentration of the initial reactants. Finally, the solution had the appearance of a turbid white suspension, which indicated the formation of silica colloidal nanoparticles. The solution was stirred using an overhead motor with a PTFE stirrer blade under sealed atmosphere for 2 h after which time the reaction came to completion. This recipe was denoted as recipe e, another six recipes, denoted as a, b, c, d, f and g, were prepared by using six different ethanol volumes of 41, 52, 63, 73, 104, 125 ml, respectively, and fixing the other reaction conditions.

### Self-assembly of SNPs into opal PC films

Batches of uniform SNPs were prepared using the SV method described in above section. Silica opal PC films for each recipe were fabricated by allowing the SNPs to settle through natural sedimentation (Mayoral et al. 1997). Specifically, samples from an unpurified silica suspension containing uniform SNPs were allowed to settle on a flat glass substrate (a beaker, basin or glass slide) through sedimentation by gravity. Once the sample solution was air-dried at room temperature (RT) or at an elevated temperature in a Gallenkamp hot-box laboratory oven, a solid opal PC film of stacked SNPs was obtained. Due to the uniformity of sphere shape and the narrow range of particle diameter size, the resulting opal films diffracted incident white light, which produced a range of spectral colours from violet to red.

### Characterisation of SNPs

Measurements of particle diameter and morphology were obtained using scanning electron microscopy and dynamic light scattering.

#### Scanning electron microscopy (SEM)

The physical characterisation of SNPs and opal films were examined using scanning electron microscopy (SEM). The SEM micrographs were captured using a Zeiss EVO 50 scanning electron microscope. Using the Java-based image processing software ImageJ, the sphere diameter size and standard deviation (SD) were calculated from the SEM micrographs. As silica is a non-conductive material, the silica samples were coated with a conductive layer (gold in this case) as part of the sample preparation process.

#### Dynamic light scattering (DLS)

A Malvern Zetasizer Nano S dynamic light scattering (DLS) device was used to measure the particle size and size distribution of the SNPs. The growth rates of the silica particles in the solution were analysed using DLS, as the technique provides an effective way to measure the particle size directly from an original silica suspension with little sample preparation required such as drying or gold coating.

## Results and discussion

Pieranski (Pieranski 1983) provided a detailed reliable recipe for the synthesising of 300 nm diameter SNPs at room temperature (RT). It has been proved that particle diameter decreases monotonically as temperature increases (Bogush et al. 1988). So in this work, firstly, uniform SNPs with a reduced size of 159 nm (average diameter) were successfully produced by using Pieranski’s recipe, but at the higher temperature of 60 °C, this recipe is the recipe e, and it was set as the standard recipe. Then, by varying the volume of ethanol and fixing the other reaction conditions relative to this standard recipe, i.e. the volume amount of TEOS/H_2_O/NH_3_ (25%) is 6 ml/3 ml/8 ml, reaction temperature is 60 °C and stirring time is 2 h, six more recipes (a, b, c, d, f and g) were produced. Each recipe was reproduced three times to determine the repeatability error in average particle diameter.

**Fig. 1:**
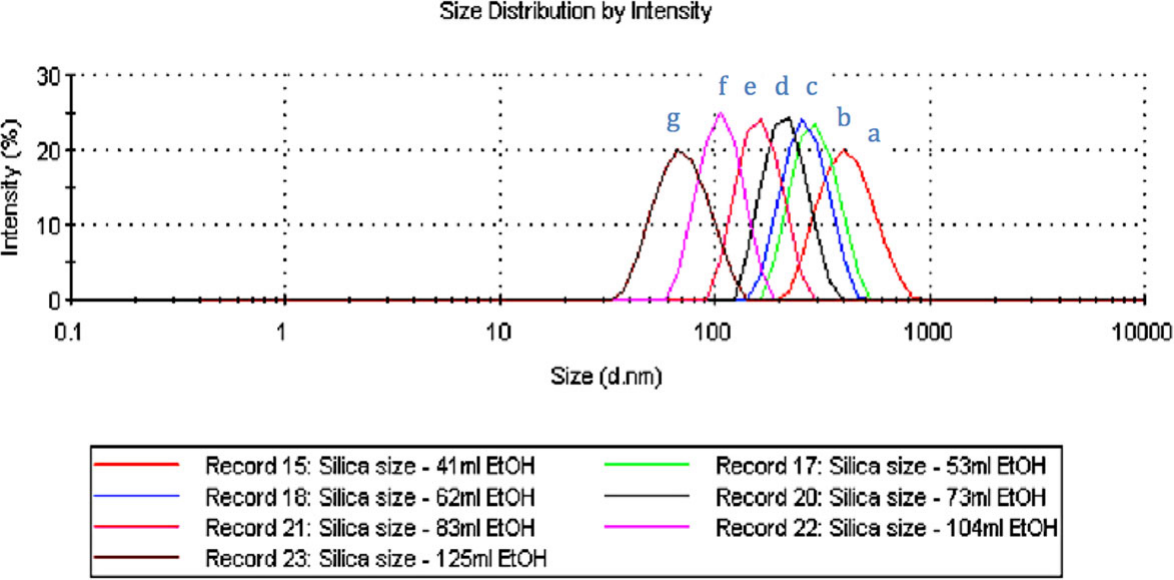
The size distributions of SNPs produced using the SV method

**Fig. 2:**
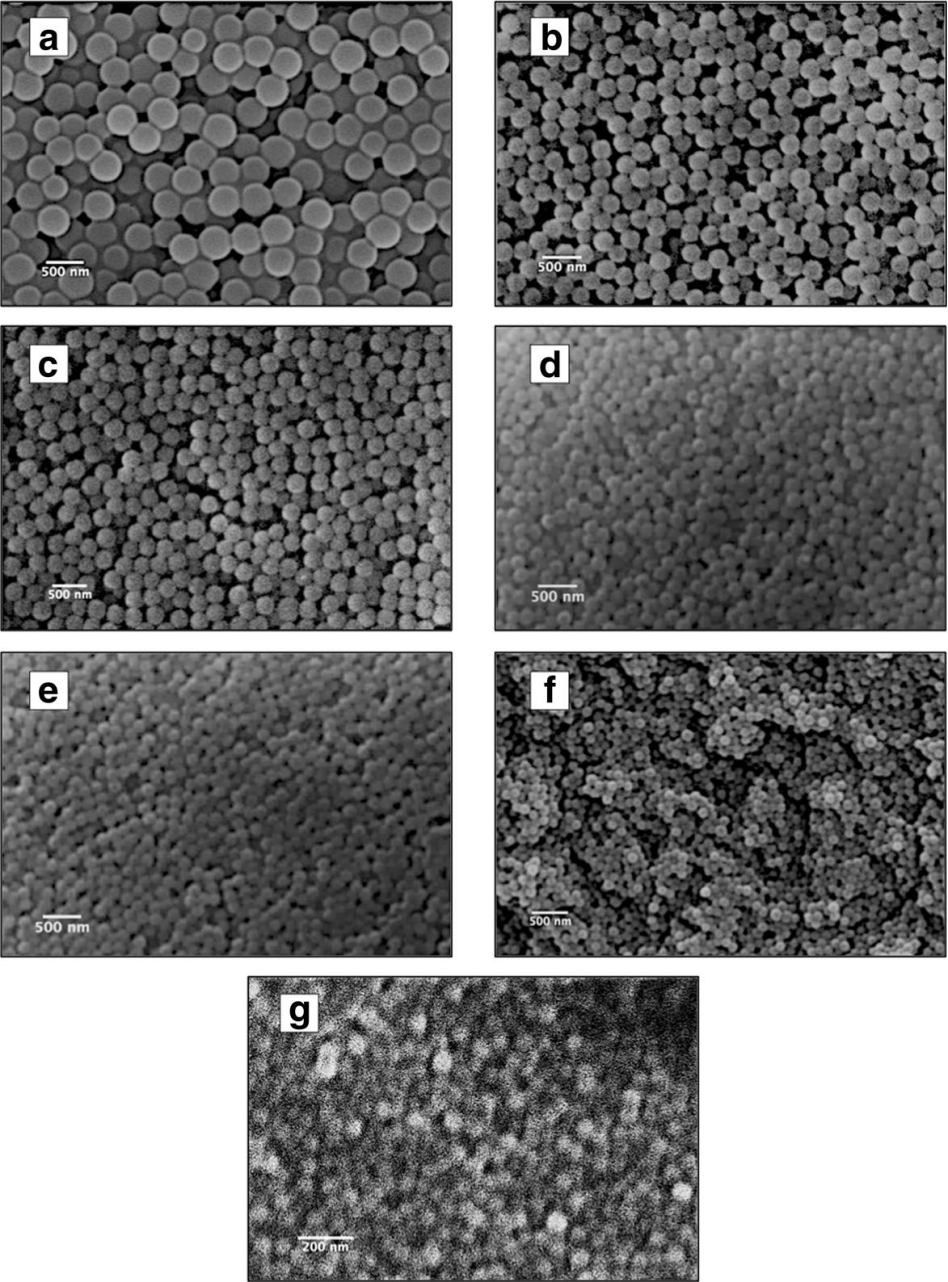
SEM images of SNPs prepared using the SV method

The particle size for each test was measured using DLS, and the final average particle diameter was calculated by averaging the results of each test. As can be seen from Table 1, the average particle diameter decreased from 398 to 73 nm as the ethanol volume was increased; however, the size distribution in terms of the PDI was smaller than 0.06. Figure 1 groups the size distributions of the SNPs from the seven recipes a–g. The normal distribution of the curve confirms the high uniformity of the prepared SNPs and the peak correlates to the average particle diameter. The results were substantiated by the data measured using ImageJ from the SEM micrographs of SNPs presented in Fig. 2. Figure 2 shows uniform SNPs with decreasing diameters when increasing volumes of ethanol were used. Based on these results, a relationship between final particle diameter and ethanol volume can be inferred.

**Table 1:** Particle diameter and PDI (in parentheses) of SNPs prepared using the SV method

Recipe number	EtOH (ml)	Test 1 diameter (nm)	Test 2 diameter (nm)	Test 3 diameter (nm)	Average diameter (nm)
a	41	397 (0.028)	402 (0.028)	395 (0.066)	398 (0.041)
b	53	282 (0.028)	292 (0.016)	288 (0.064)	287 (0.036)
c	62	257 (0.012)	239 (0.019)	275 (0.060)	257 (0.030)
d	73	207 (0.036)	204 (0.020)	214 (0.038)	208 (0.031)
e	83	161 (0.012)	157 (0.023)	158 (0.037)	159 (0.024)
f	104	101 (0.042)	104 (0.044)	97 (0.024)	101 (0.037)
g	125	79 (0.039)	68 (0.085)	72 (0.034)	73 (0.053)

### Effect of the volume of the solution on particle diameter

**Fig. 3:**
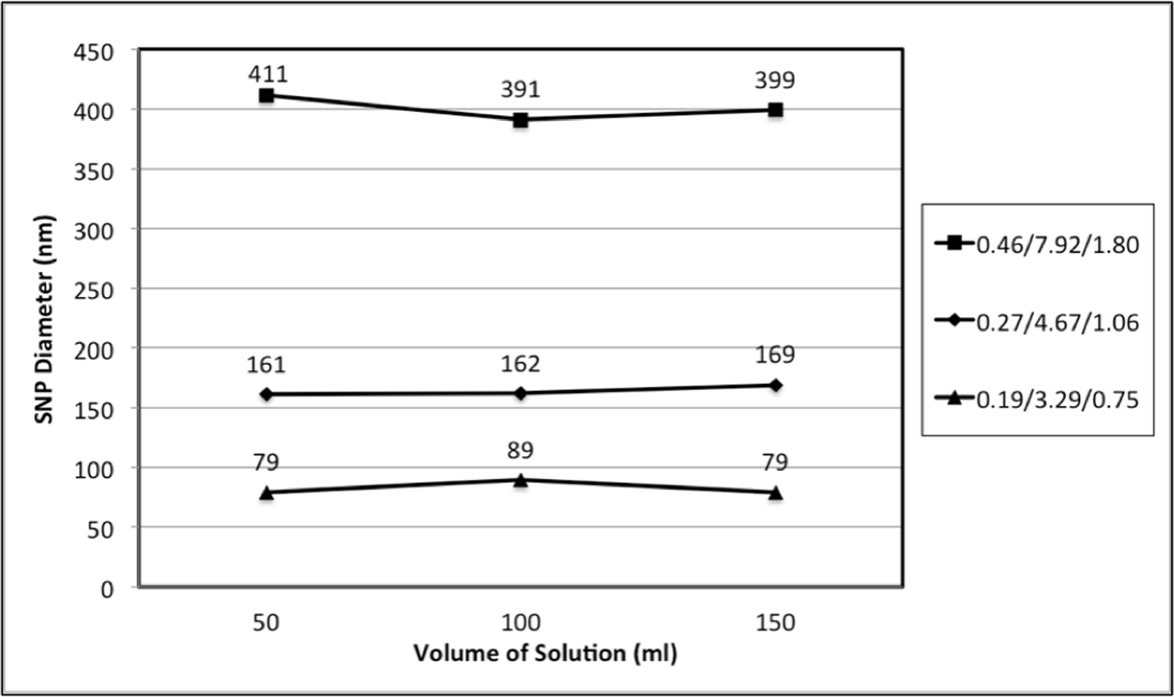
Average diameter of SNPs plotted against the total volume of solution. Three combinations of TEOS/NH_3_ (absolute)/H_2_O with differing molar ratios were calculated from recipes a, e and g, respectively

It should be noted that besides the volume of ethanol in the solution, there is another variable, the volume of total solution. Prior to analysing the effect of the volume of ethanol on particle diameter, the effect of the volume of the total solution was investigated. In order to determine whether the total volume would affect the final particle diameter, the molar concentrations of TEOS/NH_3_ (absolute)/H_2_O were calculated for recipe a, e and g. Three volumes (50, 100 and 150 ml) of solution were selected for comparison. The average particle diameters for the three selected solution volumes are plotted in Fig. 3. Particle size measurements based on the SEM images in Fig. S1 and the DLS data in Table S1 can be found in the Supporting Information. Comparison between the three sets in Fig. 3 shows that the average size of silica particles has a dramatic change (at least an 82% increase from 89 to 162 nm) for different molar concentrations, while horizontal comparison shows that particle sizes for different volumes of solution are very similar. Thus, it may be suggested that the volume of solution will have little affect on the final silica size, but the concentration of the reagents has a more significant effect.

### Effect of the volume of solvent on particle diameter

**Fig. 4:**
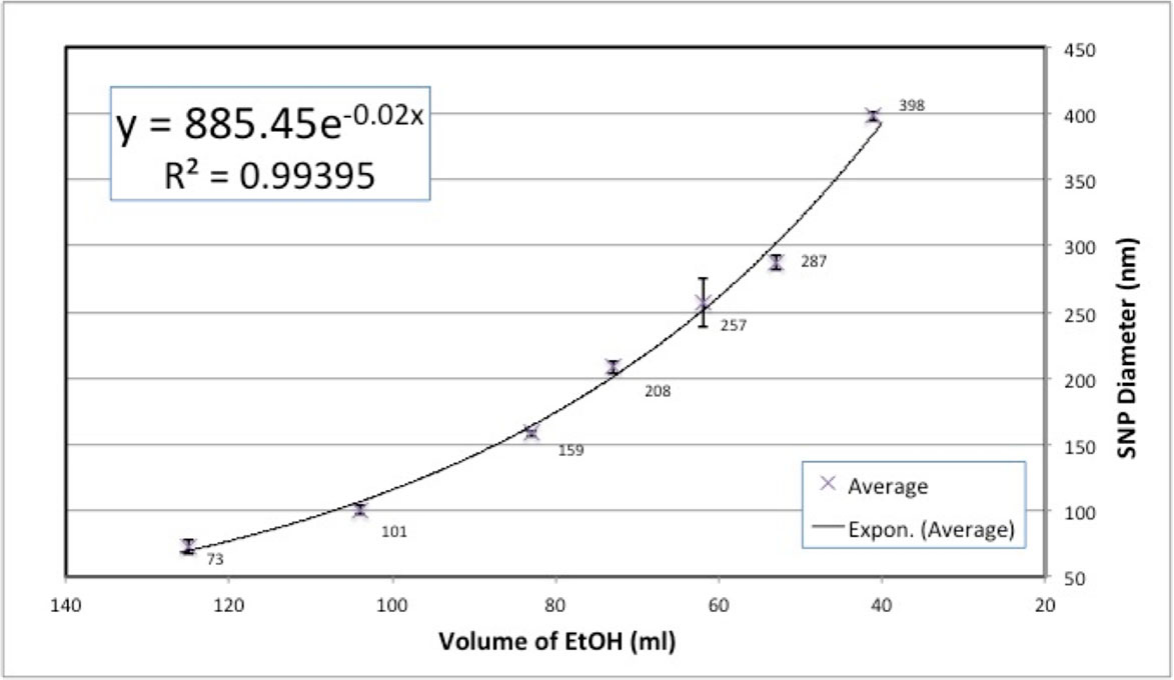
The average diameter of SNPs plotted against EtOH volume

The effect of the total volume of solution to the final SNP diameter can be assumed to be minimal (Fig. 3). Thus, the most effective variable is the volume of ethanol in the solution. In Fig. 4, the dependence of the average particle diameter on the ethanol volume is shown for the same TEOS/H_2_O/NH_3_ (25%) volume amounts of 6/3/8 ml and at the same temperature of 60 °C. An exponential trend line has been added to Fig. 4 based on the seven average diameters of the SNPs taken from Table 1, as this type of trend line gives an *R* ^2^ value of 0.99395. The max-min error bars are included in Fig. 4 in order to represent the error range for all repeated recipes. The resulting exponential expression data fit assumes that the volumes of the other reagents are fixed as given above and are written as follows:

1 $$ d=885.45 \exp \left(-0.02\left[{V}_{EtOH}\right]\right) $$

where *d* is the diameter of the SNPs in nanometers (*y*-axis in Fig. 4) and *V*_*EtOH*_ is the initial volume of ethanol added in the solution, which is given in ml (*x*-axis in Fig. 4). This fitted exponential function contains only one parameter, that of ethanol volume, which can be used to predict the diameter of the final SNPs in the range of 70 to 400 nm. It should be noted that the smallest size distribution (PDI = 2.4%) was achieved for the standard recipe e from Table 1, while the PDI value increased when used with either higher or lower volumes of ethanol (PDI = 4.1 and 5.3%). The largest PDI was 8.5% (0.085 < 0.1) in test 2 of recipe g in Table 1, but as the PDI value was smaller than 0.1, the resulting SNPs are uniform in size.

From Eq. (1), it can be seen that there is a negative correlation between the particle size and the ethanol volume, which is the smaller the ethanol volume, the larger the particle size that will be obtained. If only TEOS is considered, then the molar concentration increases from 0.19 to 0.46 M when the ethanol volume decreases from 125 to 41 ml. When the concentration of TEOS becomes larger, more reaction intermediates are produced within a unit volume during the hydrolysis of TEOS; therefore, a larger particle can be formed from the condensation of those intermediates. Bogush et al. (1988) reported a similar phenomenon at 55 °C where they found that the particle size increased from 150 to 250 nm when the molar concentration of TEOS increased from 0.1 to 0.35 M.

It is still difficult to fully explain the mechanism of this observed relationship because all of the molar concentrations of TEOS/H_2_O/NH_3_ (25%) are changed at the same time when only varying the ethanol volume in the reaction system. However, this method of varying the volume of ethanol is practical when producing uniform distributions of SNPs and the exponential equation provides a convenient prediction of the final SNP diameter.

### Effect of reaction time on final particle diameter

**Fig. 5:**
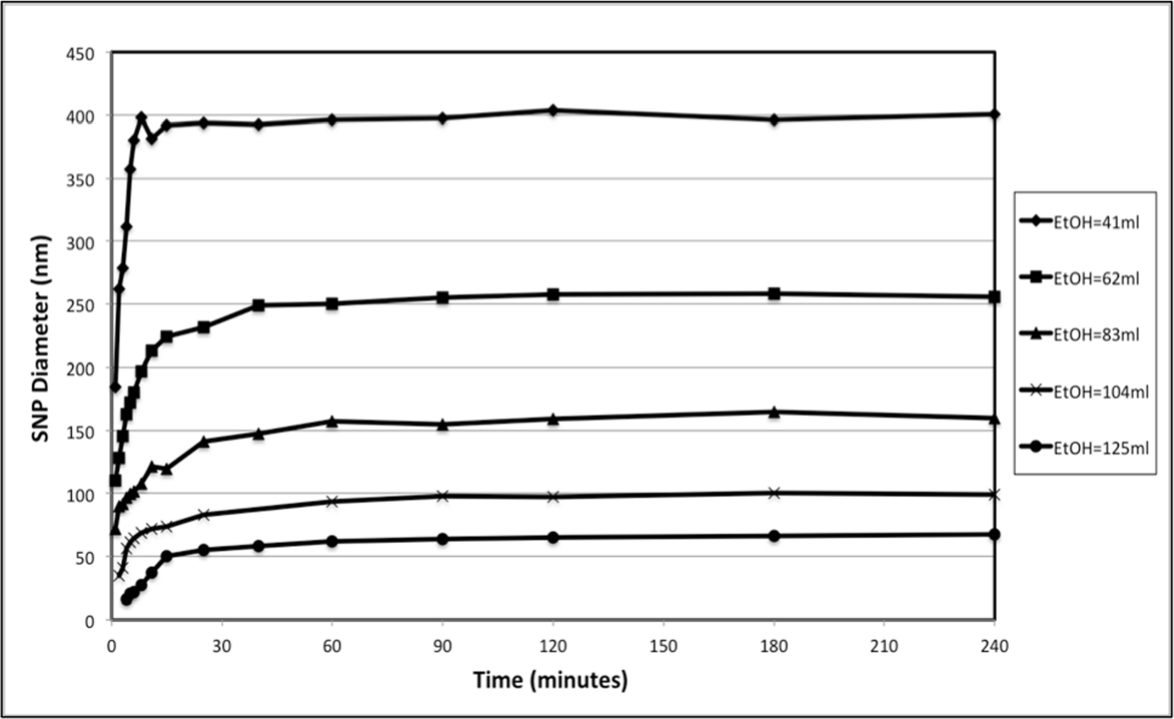
The growth of SNP diameter against reaction time

The particle diameter in solution during reaction was characterised using a dynamic light scattering (DLS) instrument. In order to measure the particle diameter at a certain time during the reaction, the reaction must be quenched prior to DLS measurement. Generally, there are three ways to stop the reaction: termination by adding trimethychlorosilane (Giesche 1994), dilution with solvent alcohol (Kim et al. 2002; Nozawa et al. 2005) and rapid removal of the reaction solution by accelerated drying of the sample (Bogush and Zukoski 1991; Chen et al. 1996b; Chen et al. 1997). In this study, for each of the five recipes in (excluding rows b and d), 0.02 ml of each reaction solution was extracted over the duration of 1 to 240 min (16 time intervals) and diluted with 1.5 ml of ethanol in order to terminate the reaction. The DLS was used to determine the particle diameter for each sample, Table S2 in the Supporting Information. These results were obtained from over 16 samples for the five experiments presented in Fig. 5.

There are two major phenomena that can be inferred from Fig. 5. Firstly, the greater the volume of ethanol used, the higher the growth rate. Secondly, regardless of the reaction rate, the growth time for the final achievable particle size was approximately 120 min (2 h). This contradicts some previous studies, which state that the reaction required between 3 and 12 h to reach completion (Bogush et al. 1988; Kim et al. 2002; Nozawa et al. 2005). This suggests a minimum time of 2 h for sample preparation, which would shorten the production time required to create mature SNPs for further application.

### Reproduction of SNPS and size prediction

Equation 1 allows the prediction of the diameter of mature SNPs. SNPs with target diameters in a range of 150–400 nm have been produced, as natural opals having silica spheres in this diameter range will give rise to structural colours (Jones et al. 1964; Sanders 1964; Darragh et al. 1976). On completion of a 2-h reaction, all of the samples of each solution were measured using the DLS and SEM techniques. The particle size and particle population dispersity can be obtained directly from the DLS analysis. The image processing software ImageJ was used to calculate the particle diameter from the SEM images. Table 2 shows the results of measured particle diameters including PDI and standard deviation (SD) determined by using DLS, SEM and those predicted from Eq. (1).

**Table 2:** SNP diameters obtained from Eq. (1), DLS and SEM; the PDI from DLS and SD from SEM are given in parentheses

EtOH volume (ml)	Equation (1) diameter (nm)	DLS		SEM	
		Diameter (nm)	PDI	Diameter (nm)	SD
41	390	397	0.041	391	54
45	360	400	0.036	393	48
47	346	350	0.111	320	47
50	326	369	0.028	343	27
53	307	282	0.028	300	28
55	295	278	0.002	291	27
57	283	288	0.040	307	28
60	267	270	0.087	267	22
62	256	259	0.050	252	22
64	246	242	0.007	254	35
67	232	249	0.025	260	27
73	206	207	0.036	210	20
78	186	181	0.016	187	24
83	168	159	0.024	173	37

**Fig. 6:**
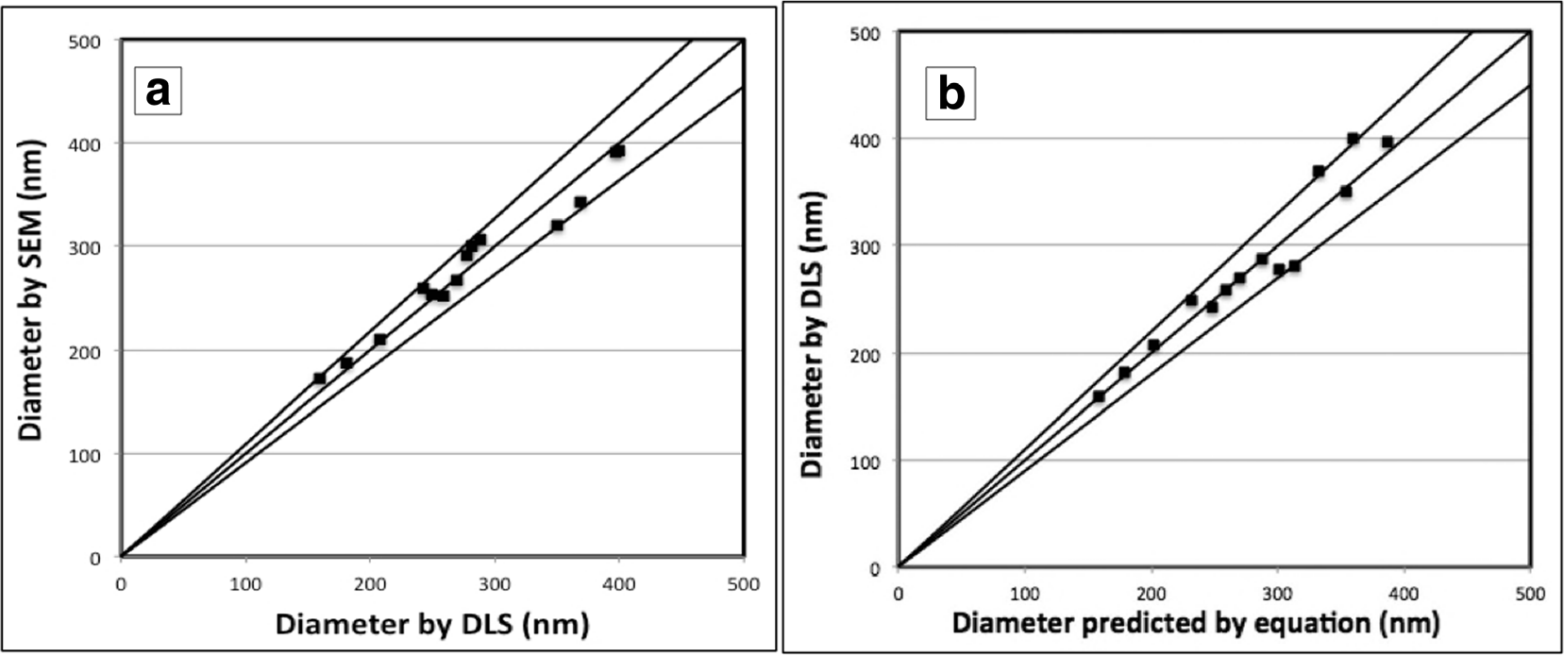
The comparison of SNP diameters between the DLS and SEM measurement techniques (**a**), DLS and Eq. (1) (**b**); perfect correlations would correspond to the *central lines*, and the two *outer lines* represent 9% and 10% deviation for **a** and **b** respectively

The results from the predicted SNP diameters from Eq. (1) and the measured sizes from DLS and SEM are displayed in Fig. 6. Firstly, as shown in Fig. 6a, the DLS measurements of the particle size were found to be in good agreement with the SEM data in terms of a deviation of ±9%. This suggests that the timesaving DLS can be used to determine the SNP diameter instead of using the SEM. In addition, Fig. 6b shows an overall agreement between the prediction of particle sizes from Eq. (1) and the corresponding values determined from the DLS technique. Figure 6b shows that all the data falls within the outer lines, which represent a ±10% error in the correlation, giving an improved result compared to the 20% correlation reported in the literature (Bogush et al. 1988). Equation (1) provides a reasonable approximation of the size prediction of SNPs. However, it should be noted that the basic reaction requirements used for this equation are the fixed TEOS/H_2_O/NH_3_ (25%) volume amounts and a temperature of 60 °C.

### Structurally coloured opal PC films using SNPs

Colloidal suspensions containing uniform SNPs were prepared using the SV method, without any purification and/or modification. These were dried at 60 °C in a Gallenkamp hot-box laboratory oven. Opal PC films were produced by sedimentation of the SNPs onto the bottom of the container over a duration of 10 min.

**Fig. 7:**
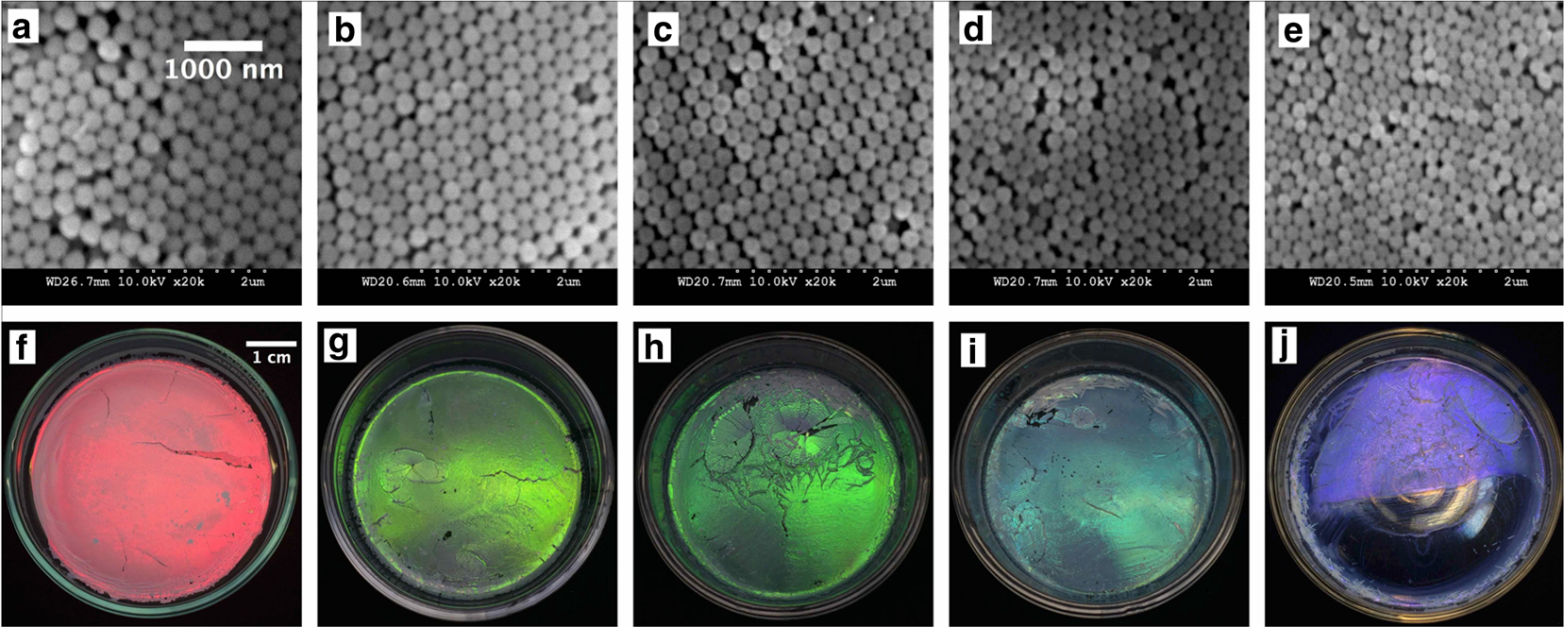
Surface SEM images (**a**–**e**) and structural colours (**f**–**j**) of artificial opal photonic crystal films self-assembled from SNP diameter of 350 nm (5.2%), 282 nm (2.8%), 270 nm (8.7%), 249 (2.5%) and 207 nm (3.6%), respectively; PDI is given in parentheses

It was observed that only those suspensions having uniform particle populations in the diameter range of 207 to 350 nm resulted in coloured opal PC films covering the full visible spectrum. The SEM images and tuneable structural colours of such film samples have been reported elsewhere (Gao et al. 2016), as seen in Fig. 7. Specifically, in Fig. 7, structural colours of red, yellow, green, cyan and violet were observed on Petri dishes from self-assembled PCs consisting of SNPs with diameters of 350, 282, 270, 249 and 207 nm, respectively. The prepared SNPs were very uniform as the PDIs were all smaller than 0.1. However, the drying of suspensions of SNPs with diameters beyond this range produced films with the same milky white opalescent appearance as observed from potch opals.

In precious opals, the relationship between colour (in terms of its peak wavelength λ) and particle diameter *d* can be described by applying a modified Bragg’s law, as shown in Eq. (2) (Tilley 2010):

2 $$ \lambda =1.633d{\left({n^2}_{eff-}{ \sin}^2{\theta}_1\right)}^{1/2}/m $$

where *n*_*eff*_ is the effective refractive index of the silica opal material, *m* is the order of diffraction and *θ*_1_ is the incident angle of the light. The longest achievable wavelength *λ*_*max*_ will occur at normal incidence, when sin*θ*_1_ = 0 and *m* = 1; thus, Eq. (2) can be rewritten as Eq. (3) (Gao et al. 2016):

3 $$ {\lambda}_{\max }=1.633{dn}_{eff} $$

According to Eq. (3), it is clear that the wavelength *λ*_*max*_ will decrease with a decrease in particle diameter *d*; thus, the colour will shift from red to blue (blue-shift) due to the decrease of particle size from 350 to 307 nm. This would explain the colour effects observed in Fig. 7.

Although the SNP diameter range of 207–350 nm of coloured artificial opals is not in agreement with the particle diameters reported in natural opals (Darragh et al. 1976; Nassau 2001) as it has been reported that the violet will appear at a particle size of 138 nm and the red appears at 241 nm, the results could at least explain the practicability of the prediction Eq. (1) to produce uniform SNPs for PC applications requiring colour display and sensing.

## Conclusions

The presented solvent varying (SV) method provides a one-step procedure that allows facile control over the diameter of the final SNPs by only varying the ethanol volume and fixing the other reaction conditions. Seven batches of SNPs with diameters ranging from 70 to 400 nm have been produced with a narrow distribution of particle diameter (PDI less than 0.1). DLS and SEM results were highly correlated with a 10% deviation, which confirmed the uniformity and diameter measurements of the produced SNPs. Intense structural colours were observed from all of the self-assembled opal films. Moreover, the DLS analysis within the 70–400 nm size range reveals that the particle growth had completed within 2 h, suggesting a minimum time for sample preparation and a shortening of production times. The reproducibility and reliability of the SV method in the specified size range allow high-quality artificial opal PCs to be fabricated and the structural colour to be tuned for further applications.

## Electronic supplementary material

ESM 1. SEM images (Fig. S1) and DLS data (Table S1) of SNPs prepared using different volume of solution, DLS data of SNPs diameter against reaction time (Table S2). These materials are available via the Internet at www.springer.com. (DOC 325 kb)
