# Supplementary material for: Facile control of silica nanoparticles using a novel solvent varying method for the fabrication of artificial opal photonic crystals
Source: J Nanopart Res. 2016 Dec 17;18(12):387. doi: 10.1007/s11051-016-3691-8 (PMC5161756; doi:10.1007/s11051-016-3691-8)
Supplement: Supplementary file 1 — SEM images (Fig. S1) and DLS data (Table S1) of SNPs prepared using different volume of solution, DLS data of SNPs diameter against reaction time (Table S2). These materials are available via the Internet at www.springer.com. (DOC 325 kb) [file 11051_2016_3691_MOESM1_ESM.doc]

Supporting Information

Facile control of silica nanoparticles using a novel solvent varying method for the fabrication of artificial opal photonic crystals

*Weihong Gao,^a^ Muriel Rigout^b^ and Huw Owens*^a^*

***^a^***School of Materials, The University of Manchester, Manchester, M13 9PL, UK

***^b^***School of Design, University of Leeds, Leeds, LS2 9JT, UK

***Corresponding Author. E-mail Address: Huw.Owens@manchester.ac.uk; Tel: +44-161-3065891


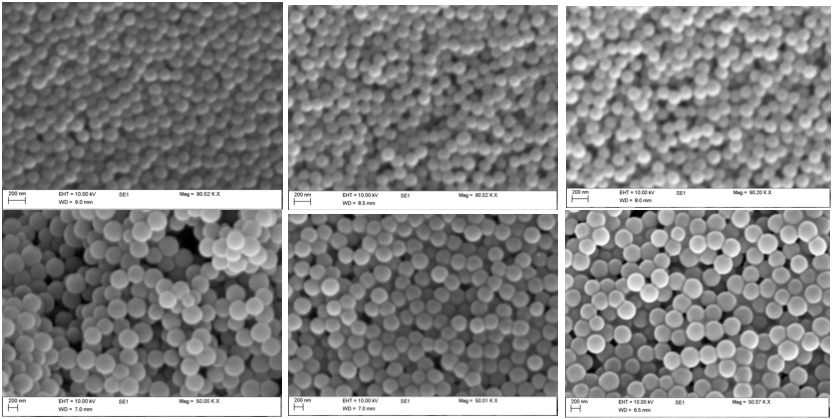


Figure S1. SEM images of SNPs prepared from two different TEOS/ NH_3_ (absolute) / H_2_O molar ratio sets, which are 0.27 / 4.67 / 1.06 for the above three images and 0.46 / 7.92 / 1.8 for bottom three images; in addition, the total volume of solution is 100 ml, 50 ml, 150 ml for left to right images of both sets.

**Table S1.** DLS measurement of SNPs prepared from TEOS/ NH_3_ (absolute) / H_2_O molar ratio of 0.19 / 3.29 / 0.75 in total solution volume of 50 ml, 100 ml, and 150 ml for the samples with record number of 1, 2, and 3, respectively. The Z-Average was treated as the average diameter of the measured SNP sample. This value was used to plot Fig. 2 in the main text.

| Record Number | Sample Name | Temperature | Z-Average | PdI | Volume Mean | Number Mean | Intensity Mean | Mean Count Rate | Percent In Size Range |
| --- | --- | --- | --- | --- | --- | --- | --- | --- | --- |
| 1 | SNP size-50ml | 24.9 | 79.05 | 0.030 | 73.31 | 64.11 | 83.12 | 340.0 | 100 |
| 2 | SNP size-100ml | 25.1 | 88.59 | 0.077 | 80.10 | 64.90 | 96.65 | 328.6 | 100 |
| 3 | SNP size-150ml | 24.9 | 78.99 | 0.047 | 72.86 | 62.95 | 83.75 | 383.2 | 100 |

**Table S2.** DLS measurement of SNPs prepared using five different ethanol volumes (Measurements were collected based on particle size for batches with a PDI smaller than 0.1, the data that does not satisfy this criterion was ignored and marked with a diagonal line).

| Record Number | Reaction Time  (Minutes) | Z-Average (d. nm) | | | | |
| --- | --- | --- | --- | --- | --- | --- |
|  |  | Volume of EtOH=41ml | Volume of EtOH=62ml | Volume of EtOH=83ml | Volume of EtOH=104ml | Volume of EtOH=125ml |
| 1 | 1 | 183 | 110 | 72 |  |  |
| 2 | 2 | 258 | 128 | 90 | 35 |  |
| 3 | 3 | 278 | 146 | 91 | 41 |  |
| 4 | 4 | 308 | 163 | 96 | 56 | 16 |
| 5 | 5 | 357 | 172 | 99 | 61 | 21 |
| 6 | 6 | 380 | 180 | 102 | 65 | 22 |
| 7 | 8 | 398 | 197 | 108 | 69 | 27 |
| 8 | 11 | 381 | 213 | 121 | 72 | 37 |
| 9 | 15 | 392 | 224 | 120 | 74 | 50 |
| 10 | 25 | 394 | 232 | 141 | 83 | 55 |
| 11 | 40 | 393 | 249 | 147 |  | 58 |
| 12 | 60 | 396 | 250 | 157 | 94 | 62 |
| 13 | 90 | 397 | 255 | 155 | 98 | 64 |
| 14 | 120 | 404 | 257 | 159 | 97 | 65 |
| 15 | 180 | 396 | 258 | 164 | 100 | 66 |
| 16 | 240 | 401 | 256 | 160 | 99 | 67 |
